# Commercial Price Variation for 11 Outpatient-Based Psychiatric Services

**DOI:** 10.1001/jamanetworkopen.2025.52939

**Published:** 2026-01-20

**Authors:** Kyle King, Joshua J. Skydel, Joseph S. Ross, Joshua D. Wallach

**Affiliations:** 1 Yale School of Medicine, New Haven, Connecticut; 2 Section of Rheumatology, Allergy, and Immunology, Yale School of Medicine, New Haven, Connecticut; 3 Collaboration for Regulatory Rigor, Integrity, and Transparency, Yale School of Medicine, New Haven, Connecticut; 4 Section of General Internal Medicine, Yale School of Medicine, New Haven, Connecticut; 5 Department of Health Policy and Management, Yale School of Public Health, New Haven, Connecticut; 6 Department of Epidemiology, Rollins School of Public Health, Atlanta, Georgia

## Abstract

This cross-sectional study evaluates commercial price variation for common outpatient psychiatric services in the US.

## Introduction

Prices for outpatient medical services in the US have increased over the past decade^1^ and represent a significant barrier to care, particularly for patients requiring psychiatric services.^2^ Individuals with mental illness are more likely to experience financial insecurity, and nearly one-third report delaying or forgoing treatment due to cost.^3^ Although previous research has identified price variability for numerous hospital-based services,^4,5^ little is known about its extent in psychiatric services. Therefore, we evaluated commercial price variation for common outpatient psychiatric services.

## Methods

In this cross-sectional study, we identified prices for 11 commonly billed outpatient psychiatric services using *Current Procedural Terminology* codes (eMethods in Supplement 1). We searched the Turquoise Health database to identify rates negotiated by the 4 largest US private payers (Aetna, Blue Cross Blue Shield, Cigna, and UnitedHealthcare) for each service as of June 2025, as well as the list, cash, and Medicare prices for hospitals reporting at least 1 commercial payer–negotiated rate (eTable in Supplement 1). This study followed the STROBE reporting guideline; it did not constitute human participant research and was thus exempt from ethics review per the Common Rule.

We adjusted commercial payer–negotiated rates, list prices, and cash prices using the geographic adjustment factor to account for geographic differences and excluded prices outside the 1st and 99th percentiles to reduce outlier effects.^5^ For each service, we calculated the median, IQR, 10th percentile, and 90th percentile of the adjusted commercial payer–negotiated rate and the ratio of median commercial payer–negotiated rate to median list, cash, and Medicare price. We assessed within-hospital variation by calculating the median ratio of the 90th to 10th percentile of commercial payer–negotiated rates within each hospital and between-hospital variation by calculating the ratio of the 90th to 10th percentile of rates across all hospitals. We stratified rates by payer, calculated the median rate and 10th to 90th percentile range within each group, and assessed differences using Kruskal-Wallis tests. Two-tailed *P* < .05 was considered statistically significant. Analyses were performed using R, version 4.2.3.

## Results

We identified 94 228 unique commercial payer-negotiated rates (median per service, 216 [IQR, 155-342]) reported by 1398 hospitals across 49 US states and the District of Columbia. Across all psychiatric services, commercial payer–negotiated rates were a median of 1.37 (IQR, 1.02-1.47) times the Medicare rate, 0.59 (IQR, 0.38-0.85) times the list price, and 1.12 (IQR, 0.87-2.13) times the cash price for the same service. There was variation in commercial payer–negotiated rates both within (median within-hospital ratio, 1.15 [IQR, 1.14-1.16]) and between hospitals (median between-hospital ratio, 4.16 [IQR, 3.82-6.63]) for the same service (Table 1).

**Table 1.** Characteristics of Commercial Payer-Negotiated Rates for 11 Outpatient-Based Psychiatric Services

Service (*CPT* code)	No. of rates	No. of hospitals	GAF-normalized commercial payer-negotiated rate, $			Ratio of median rate to Medicare prices	Within-hospital ratio^a^	Between-hospital ratio^b^
			Median (IQR)	10th percentile	90th percentile			
Psychotherapy, 30 min with patient (90832)	9029	1020	118 (81-216)	69	379	1.08	1.15	5.45
Psychotherapy, 45 min with patient (90834)	9054	1014	170 (112-243)	96	399	1.17	1.14	4.16
Psychotherapy, 60 min with patient (90837)	8517	980	223 (155-382)	134	432	1.48	1.11	3.22
Family or couples psychotherapy without the patient present (90846)	8280	920	188 (122-271)	99	429	1.35	1.15	4.33
Family or couples psychotherapy with the patient present (90847)	8727	960	205 (127-285)	105	432	1.45	1.15	4.11
Group psychotherapy (other than of a multiple-family group) (90853)	11 540	980	95 (49-214)	30	250	1.51	1.16	8.33
Psychiatric diagnostic evaluation (without medical services) (90791)	8741	1000	216 (161-342)	141	496	1.37	1.19	3.52
Psychiatric diagnostic evaluation with medical services (90792)	7107	916	232 (176-374)	148	504	1.39	1.13	3.41
Electroconvulsive therapy (90870)	7859	893	532 (249-1021)	133	1593	1.31	1.18	11.98
Transcranial magnetic stimulation treatment, initial (90867)	7711	701	476 (316-785)	297	1225	1.67	1.16	4.12
Transcranial magnetic stimulation treatment, subsequent sessions (90868)	7663	694	352 (299-707)	154	1201	1.21	1.12	7.80

Abbreviations: *CPT*, *Current Procedural Terminology*; GAF, geographic adjustment factor.

^a^ Within-hospital variation: the median ratio of the 90th to 10th percentile commercial payer–negotiated rates within each hospital.

^b^ Between-hospital variation: the ratio of the 90th to 10th percentile of rates across all hospitals.

Median commercial payer–negotiated rates varied significantly across parent payers for all psychiatric services (Table 2). For 8 of 11 services, the highest median rate was negotiated by Cigna. All insurers showed substantial variation in negotiated rates for services, with Blue Cross Blue Shield having the widest rate range for 6 of 11 codes.

**Table 2.** Comparison of Commercial Payer–Negotiated Rates for 11 Outpatient-Based Psychiatric Services

Commercial payer	Median (IQR) commercial payer-negotiated rate, $	Commercial payer-negotiated rate, $			*P* value^a^
		10th Percentile	90th Percentile	Difference	
**Psychotherapy, 30 min with patient (*CPT* code 90832)**					
BCBS	153 (81-285)	70	438	368	<.001
Cigna	104 (100-142)	70	271	201	
Aetna	104 (81-158)	71	276	205	
UnitedHealthcare	122 (77-160)	64	259	195	
**Psychotherapy, 45 min with patient (*CPT* code 90834)**					
BCBS	170 (109-303)	95	474	379	<.001
Cigna	190 (136-191)	100	297	197	
Aetna	185 (118-205)	101	327	226	
UnitedHealthcare	146 (105-192)	92	307	215	
**Psychotherapy, 60 min with patient (*CPT* code 90837)**					
BCBS	212 (156-343)	135	474	339	<.001
Cigna	339 (190-382)	147	382	235	
Aetna	258 (158-382)	141	382	241	
UnitedHealthcare	174 (146-278)	114	384	270	
**Family or couples psychotherapy without the patient present (*CPT* code 90846)**					
BCBS	191 (121-307)	98	474	376	<.001
Cigna	256 (145-256)	105	309	204	
Aetna	205 (130-307)	110	314	204	
UnitedHealthcare	148 (115-205)	97	319	222	
**Family or couples psychotherapy with the patient present (*CPT* code 90847)**					
BCBS	217 (121-315)	114	309	195	<.001
Cigna	262 (153-262)	114	309	195	
Aetna	206 (133-262)	113	346	233	
UnitedHealthcare	157 (125-234)	99	354	255	
**Group psychotherapy (other than of a multiple-family group) (*CPT* code 90853)**					
BCBS	91 (36-188)	28	296	268	<.001
Cigna	214 (81-214)	36	214	178	
Aetna	115 (53-214)	44	214	170	
UnitedHealthcare	74 (53-214)	29	210	181	
**Psychiatric diagnostic evaluation (without medical services) (*CPT* code 90791)**					
BCBS	223 (172-350)	148	510	362	<.001
Cigna	232 (170-318)	137	443	306	
Aetna	209 (155-342)	143	488	345	
UnitedHealthcare	190 (148-341)	132	501	369	
**Psychiatric diagnostic evaluation with medical services (*CPT* code 90792)**					
BCBS	232 (189-377)	151	488	337	<.001
Cigna	250 (177-364)	151	586	435	
Aetna	238 (166-379)	151	536	385	
UnitedHealthcare	212 (155-375)	137	504	367	
**Electroconvulsive therapy (*CPT* code 90870)**					
BCBS	560 (244-1140)	131	1593	1462	<.001
Cigna	540 (376-1067)	161	1909	1748	
Aetna	373 (184-540)	126	903	777	
UnitedHealthcare	510 (348-577)	164	1716	1552	
**Transcranial magnetic stimulation treatment; initial (*CPT* code 90867)**					
BCBS	623 (375-933)	302	1325	1023	<.001
Cigna	501 (316-727)	316	1127	811	
Aetna	316 (312-374)	284	638	354	
UnitedHealthcare	316 (314-629)	291	1462	1171	
**Transcranial magnetic stimulation treatment; subsequent sessions (*CPT* code 90868)**					
BCBS	484 (247-933)	90	1325	1235	<.001
Cigna	497 (316-704)	299	1127	828	
Aetna	316 (297-316)	179	527	348	
UnitedHealthcare	316 (302-565)	280	1462	1182	

Abbreviations: BCBS, Blue Cross Blue Shield; *CPT*, *Current Procedural Terminology*.

^a^ Kruskal-Wallis tests comparing payers.

## Discussion

This cross-sectional study found wide variation in commercial payer–negotiated rates for 11 hospital-based psychiatric services, with within- and between-hospital price ratios of 1.15 and 4.16, respectively. Although commercial payer–negotiated rates were generally comparable with cash prices and averaged 1.37 times the Medicare rate for the same service, substantial variation was observed across the 4 largest commercial payers, potentially reflecting differences in payer and hospital market shares.^6^ These patterns, consistent with other areas of health care, such as urology and breast cancer,^4,5^ suggest that individuals with the same conditions may encounter different costs for psychiatric services depending on site of care and insurer, which may interact with out-of-pocket costs to influence care-seeking behavior.

Our analyses are limited by inconsistent compliance with price reporting requirements and the resulting uncertainty over whether price variation differs between compliant and noncompliant hospitals, although this risk may be small.^5^ Our analyses cannot determine the reasons for price differences and do not capture nonhospital outpatient psychiatric care. Future research should explore the mechanisms underlying price variation, which may inform future policy decisions.

## Notes

Supplement 1.
eMethods.
**eTable.** Outpatient-Based Psychiatric Service *Current Procedural Terminology* Codes and Descriptions

Supplement 2.
Data Sharing Statement
