# Supplementary material for: Commercial Price Variation for 11 Outpatient-Based Psychiatric Services
Source: JAMA Netw Open. 2026 Jan 20;9(1):e2552939. doi: 10.1001/jamanetworkopen.2025.52939 (PMC12820733; doi:10.1001/jamanetworkopen.2025.52939)
Supplement: Supplement 1. — eMethods. eTable. Outpatient-Based Psychiatric Service Current Procedural Terminology Codes and Descriptions [file jamanetwopen-e2552939-s001.pdf]

Supplementary Online Content

King K, Skydel JJ, Ross JS, Wallach JD. Commercial price variation for 11 outpatient-based psychiatric services. *JAMA Netw Open.* 2026;9(1):e2552939. doi:10.1001/jamanetworkopen.2025.52939

eMethods.
eTable. Outpatient-Based Psychiatric Service *Current Procedural Terminology* Codes and Descriptions

This supplementary material has been provided by the authors to give readers additional information about their work.

© 2026 King K et al. *JAMA Network Open.*

eMethods.

Identification of outpatient-based psychiatric services

We identified prices for 11 commonly billed outpatient psychiatric services using Current Procedural Terminology (CPT) codes. We selected 6 of these services because the Center for Medicare & Medicaid Services (CMS) designated them as “shoppable”, requiring hospitals to report their pricing data. We selected the remaining 5 services because the American Psychiatric Association identified them as commonly covered services.

We searched Turquoise Health to identify rates negotiated by the four largest US private payers (Aetna, Blue Cross Blue Shield, Cigna, and UnitedHealthcare) for each service as of June 2025, as well as the list, cash, and Medicare prices for hospitals reporting at least one commercial payer-negotiated rate. Turquoise Health is a third-party data platform that compiles pricing information from publicly available hospital machine-readable files, which hospitals are required to provide to comply with the CMS Hospital Price Transparency Final Rule, effective January 2021. This regulation mandates that hospitals publicly post standard charge information, including negotiated rates, in a machine-readable format. Turquoise Health collects and validates these data, which have been used to support numerous research publications. The database includes information from over 5,700 hospitals and is updated regularly, with negotiated rates updated monthly and hospital rates updated at least annually according to the frequency of new rate publications. In Maryland, average pricing for hospital-based healthcare services is regulated by the Maryland Health Services Cost Review Commission (HSCRC) rather than negotiated between hospitals and individual payers. Although rates differ across hospitals and are posted on chargemasters, they are not indexed to specific payers or insurance plans. As a result, Turquoise Health data seldom report payer-specific rates for Maryland hospitals

For each CPT code, we filtered results in Turquoise Health by Parent Payer Name (‘Blue Cross Blue Shield’, ‘Cigna’, ‘Aetna’, and ‘United HealthCare’), Payer Class (‘Commercial’), Code (only relevant codes), CPT Category, Modifier (‘No Modifier’), Hospital Type (‘Short Term Acute Care’, ‘Psychiatric Hospital’), Place of Service (‘Outpatient’, ‘Inpatient and Outpatient’), and Negotiated Type (‘Fee Schedule’).

eTable. Outpatient-Based Psychiatric Service *Current Procedural Terminology* Codes and Descriptions

***Current Procedural Terminology* Code**	**CMS Code**	**Description of Service**	**CMS Shoppable Service**
90832	Psytx w pt 30 minutes	Psychotherapy, 30 minutes with patient	Yes
90834	Psytx w pt 45 min	Psychotherapy, 45 minutes with patient	Yes
90837	Psytx w pt 60 minutes	Psychotherapy, 60 minutes with patient	Yes
90846	Family psytx w/o pt 50 min	Family or couples psychotherapy without the patient present	Yes
90847	Family psytx w/pt 50 min	Family or couples psychotherapy with the patient present	Yes
90853	Group psychotherapy	Group psychotherapy (other than of a multiple-family group)	Yes
90791	Psych diagnostic evaluation	Psychiatric diagnostic evaluation (without medical services)	No
90792	Psych diag eval w/ med srvcs	Psychiatric diagnostic evaluation with medical services	No
90870	Electroconvulsive Therapy (ECT)	Electroconvulsive therapy (ECT)	No
90867	Tcranial magn stim tx plan	Transcranial Magnetic Stimulation (TMS) treatment; initial	No
90868	Tcranial magn stint x deli	TMS treatment; subsequent sessions	No

© 2026 King K et al. *JAMA Network Open.*
