# Supplementary material for: Commercial Price Variation for 11 Outpatient-Based Psychiatric Services
Source: JAMA Netw Open. 2026 Jan 20;9(1):e2552939. doi: 10.1001/jamanetworkopen.2025.52939 (PMC12820733; doi:10.1001/jamanetworkopen.2025.52939)
Supplement: Supplement 2. — Data Sharing Statement [file jamanetwopen-e2552939-s002.pdf]

Data Sharing Statement

King. Commercial Price Variation for 11 Outpatient-Based Psychiatric Services. *JAMA Netw Open*. Published January 20, 2026. doi:10.1001/jamanetworkopen.2025.52939

Data
Data available: No

Additional Information
Explanation for why data not available: Data are available from Turquoise Health
